# Prevalence of Intestinal Parasitic Infections among Children under Five Years of Age with Emphasis on *Schistosoma mansoni* in Wonji Shoa Sugar Estate, Ethiopia

**DOI:** 10.1371/journal.pone.0109793

**Published:** 2014-10-08

**Authors:** Yirgalem G/hiwot, Abraham Degarege, Berhanu Erko

**Affiliations:** Aklilu Lemma Institute of Pathobiology, Addis Ababa University, Addis Ababa, Ethiopia; Technion-Israel Institute of Technology, Israel

## Abstract

Intestinal parasite infections are major public health problems of children in developing countries causing undernutrition, anemia, intestinal obstruction and mental and physical growth retardation. This study was conducted to assess the prevalence of intestinal helminthic infections among children under five years of age with emphasis on *Schistosoma mansoni* in Wonji Shoa Sugar Estate, Ethiopia. A cross-sectional parasitological survey was conducted in under-five children living in Wonji Shoa Sugar Estate Ethiopia, April, 2013. Stool samples were collected and examined for intestinal parasites using single Kato-Katz and single Sodium acetate-acetic acid-formalin (SAF) solution concentration methods. Out of 374 children examined using single Kato-Katz and single SAF-concentration methods, 24.3% were infected with at least one intestinal parasite species. About 10.4%, 8.8%, 4.6%, 2.9%, 1.6% and 0.8% of the children were infected with *Hymenolepis nana*, *Schistosoma mansoni*, *Ascaris lumbricoides*, *Trichuris trichiura*, *Enterobius vermicularis* and hookworm, respectively. Prevalence of double, triple and quadruple intestinal helminthic infection was 6.4%, 0.54% and 1.1%, respectively. A significant increase in prevalence of *S. mansoni* (8.3% versus 3.2%) and *T. trichiura* (2.7% versus 0.5%) infection was observed when determined via the single Kato-Katz method compared to the prevalence of the parasites determined via the single SAF-concentration method. On the other hand, the single SAF-concentration method (9.1%) revealed a significantly higher prevalence of *H. nana* infection than the single Kato-Katz (1.6%) does. In conclusion, intestinal helminths infections particularly *S. mansoni* and *H. nana* were prevalent in under-five children of Wonji Shoa Sugar Estate. Including praziquantel treatment in the deworming program as per the World Health Organization guidelines would be vital to reduce the burden of these diseases in areas where *S. mansoni* and *H. nana* infections are prevalent among under-fives. Kato-Katz can be used in estimating the prevalence of *S. mansoni* and other helminth infections.

## Introduction

Parasitic infections caused by helminths and protozoa are the major causes of human diseases in most countries of the tropical region. It is estimated that about 3.5 billion people in the world are infected with intestinal parasites, of whom 450 million are ill [1], [2]. Majority of these cases are children [2]. About 1.45 billion people in the world were infected with Soil-Transmitted Helminths (STHs) and 5.19 million show associated morbidity in 2010 [3], [4]. The estimated disease burden due to schistosomiasis was 3.31 million during 2010 [4]. Out of 1.45 billion infections due to STHs, 438.9 were infected with hookworm, 819.0 million with *A. lumbricoides* and 464.6 million with *T. trichiura* [3]. STHs are the second leading cause of mortality in children of age less than 6 years who live in Africa [5].

Majority of the intestinal parasitic infections are attributed to STHs and *Schistosoma* species. It is estimated that more than a billion people in the world are infected with schistosomes and STHs, most of whom suffer from associated severe morbidity [6]. Human schistosomiasis is of considerable public health importance and mainly affects individuals living in developing countries where water resources allow development of snails and poor sanitation facilitates infection. It is estimated that around 249 million people are infected with *Schistosoma* worldwide, many of whom live in sub-Saharan Africa [7].

Intervention against intestinal helminth infections is based on regular anti-helminthic treatment, improved water supply and sanitation and health education [8]. In developing countries, however, control measures are difficult to implement due to clear water, sanitation and education problems. As a result, intestinal helminths infection remains a significant health problem in developing regions. Furthermore, the construction of dams for the purpose of irrigation and hydroelectric power has created new areas of transmission, which intensified community level infection by *S. mansoni* in children living in Africa [9].

In Ethiopia, intestinal parasitic infections are highly prevalent, being the first or second most predominant causes of outpatient morbidity in the country [10], [11], [12]. Although the prevalence rates of individual parasite infections vary considerably in different parts of the country, most studies showed a higher prevalence of *A. lumbricoides* followed by *T. trichiura* and hookworms [10], [11], [12]. Most previous studies conducted in Ethiopia focused on school age children and only few studies reported intestinal parasitic infections among under-fives [13], [14], [15]. Thus, the current study was undertaken to assess the prevalence of intestinal helminths infection with emphasis on *S. mansoni* among children under five years of age in Wonji Sugar Estate, Ethiopia.

## Materials and Methods

### Study area and population

The study was conducted in under-five children living in Wonji Shoa Sugar Estate, Ethiopia, April, 2013. Wonji lies at the down-stream of the Koka Dam in the Awash River basin. It is located in East Shoa zone of the Oromia Region in the central rift valley of Ethiopia, 110 km southeast of Addis Ababa. The altitude of Wonji is between 1510 and1560 meter above sea level. The bimodal rainfall pattern of the area does not exceed 850 mm per year and the average temperature ranges from 17 to 30°C. The Awash River passes through the Wonji plain that is used as a source of irrigation and also water for gardening and washing. The study participants were children whose age was between six months and 5 years. Children with serious health problem and who took antihelminthic drugs within one month before screening were excluded from the study.

### Stool collection and examination

Almost all parents or guardians living in camps of the Wonji village were informed by health extension workers to bring their children of age 6 months to 5 years to a health post located in the village. More than 95% of the parents or guardians informed showed up in the health post with their children. Parents or guardians of each child were then asked to provide about 5 gm of fresh stool sample of the study child. More than 95% of the parents or guardians asked provide stool samples of their children. Some portion of the specimen was processed using Kato-Katz thick smear technique [16]. Qualitative examination for different helminths species and egg count for hookworm was performed within 45 minutes of specimen preparation in the study area. Quantitative examination for other helminths species was made at Aklilu Lemma Institute of Pathobiology, Addis Ababa University. About 2 gm of the specimen was put in plastic vial containing 10 ml sodium acetate-acetic acid-formalin (SAF) solution and was processed at Aklilu Lemma Institute of Pathobiology, Addis Ababa University for examination using sodium acetate-acetic acid -concentration method [17].

### Data analysis

Data were entered into computer and analyzed using STATA11 software. Z-test was used to test differences in prevalence of intestinal helminth infection determined between the single SAF-concentration method and the single Kato-Katz method. Pearson-chi square test was used to test for association intestinal helminth infection with children age and sex. One way ANOVA test and t-tests were used to test difference of mean egg per gram of intestinal helminths among the different age and sex groups respectively. Statistical values were considered significant when p<0.05.

### Ethical clearance

This study was ethically approved by Institutional Review Board (IRB) of Aklilu Lemma Institute of Pathobiology, Addis Ababa University before being implemented. Only children, whose parents were voluntary for their participation, were involved in the study. Oral consent was obtained from parents or guardians of the children. As the study population was mainly illiterate the IRB indorsed oral consenting of the parents or guardians of the children. Seeing that the study involved minimal risk the committee did not required tape recording or any other form of preserving the consenting processes. Children found positive for intestinal parasites were treated with appropriate drugs by physicians from Wonji Sugar Estate Hospital free of charges.

## Result

### Prevalence of intestinal helminth infections

A total of 374 under five children (mean age  = 3.3 years, 52.14% males) participated in this study. Out of 374 children examined using single Kato-Katz and single SAF-concentration method, 24.3% (91) had one or more intestinal helminths. The predominant intestinal helminth diagnosed was *H. nana* 10.4% (39) followed by *A. lumbricoides* 4.6%, (17), *T.trichiura* 2.9% (11), *E. vermicularis* 1.6% (6) and hookworm 0.8% (3). *S. mansoni* was observed in 8.8% (33) of the children.

Prevalence of intestinal helminth infections showed significant association with age (p<0.05). The prevalence of *S. mansoni* infection increased with an increase in age. However, difference in prevalence of intestinal helminth infection was not significant between males and females (Table 1).

**Table 1:** Prevalence of intestinal helminth infections (%) among under five years children as determined using single Kato Katz and single SAF concentration methods in Wonji Sugar Estate, Ethiopia, 2013.

Age	Number	Sm	Al	Tt	Ev	Hw	Hn	Any
in years	examined							helminth
≤1	42	2.4	2.4	0.0	0.0	0.0	2.4	7.1
1.1–2.0	71	4.2	2.8	4.2	2.8	0.0	9.9	19.7
2.1–3.0	73	2.7	9.6	4.1	1.4	1.4	15.1	28.8
3.1–4.0	89	7.9	7.9	4.5	2.2	1.1	12.4	30.3
4.1–5.0	99	20.2	0.0	1.0	1.0	1.0	9.1	26.3
Total	374	8.8	4.6	2.9	1.6	0.8	10.4	24.3
χ^2^		23.4	12.2	4.1	1.8	1.4	5.2	10.3
*p*		0.00	0.01	0.39	0.77	0.85	0.27	0.04
**Sex**								
Female	179	6.7	6.1	3.3	2.2	1.1	10.6	23.5
Male	195	10.8	3.8	2.6	1.0	0.5	10.3	25.1
χ^2^		1.9	2.0	0.2	0.9	0.4	0.0	0.1
*p*		0.17	0.16	0.65	0.35	0.51	0.91	0.71

*Al  =  A. lumbricoides, Tt  =  T. trichiura, Hw*  =  Hookworm species *Sm  =  S. mansoni, Hn  =  H. nana Ev  =  E. vermicularis* Any helminth  =  infected with at least one intestinal helminth species.

NB: Children positive for intestinal helminth by either the Kato-Katz or the SAF method were considered as positive.

Prevalence of intestinal helminth infection among the study participants was 15.5% (58) when estimated by single Kato-Katz method and 16% (60) when the single SAF-concentration method was used. Prevalence of *S. mansoni* infection was significantly (z = 5.60, p<0.01) higher when determined using the single Kato-Katz method (8.3%) than when the single SAF-concentration method was used (3.2%). The prevalence of *T. trichiura* infection was also significantly (z = 6.03, p<0.01) higher when determined using the single Kato-Katz method (2.7%) than when the single SAF-concentration method was used (0.5%). However, a significantly (z = 11.56, p<0.01) greater prevalence of *H. nana* was observed via the SAF-concentration method (9.1%) than the single Kat-Katz method (1.6%). Prevalence of other intestinal helminth species was comparable when determined using the single Kato-Katz and the single SAF-concentration methods (Table 2).

**Table 2:** Prevalence of intestinal helminthes infections as determined by Kato-Katz and SAF^*^ methods among under five years of age in Wonji Sugar Estate, Ethiopia, 2013.

Parasite species	Kato-Katz	SAF^*^	Either Kato-Katz or SAF^*^
	Number	number	number positive (%)
	positive (%)	positive (%)	
*S. mansoni*	31 (8.3%)	12 (3.2%)	33 (8.8%)
*A. lumbricoides*	13 (3.5%)	12 (3.2%)	17 (4.6%)
*T. trichiura*	10 (2.7%)	2 (0.5%)	11 (2.9%)
*H. nana*	6 (1.6%)	34 (9.1%)	39 (10.4%)
Hookworm	3 (0.8%)	0 (0%)	3 (0.8%)
*E. vermicularis*	5 (1.3%)	3 (0.8%)	6 (1.6%)
Double infections	8 (2.1%)	3 (0.8%)	24 (6.4%)
Triple infections	1 (0.3%)	0 (0%)	2 (0.54%)
Quadruple infections	0 (0%)	0 (0%)	4 (1.07%)
Any helminth infection	58 (15.5%)	60 (16%)	91 (24.3%)

SAF^*^: Sodium acetate-acetic acid-formalin solution.

Out of the 374 children examined based on the Kato-Katz and SAF-concentration methods, 6.4% were infected with 3 different intestinal helminth species, 0.54% were infected with 2 different intestinal helminth species and 1.1% were infected with four different intestinal helminth species. However, infection with four different helminth species was not seen when the specimen was examined using the single Kato Katz or single SAF-concentration method alone. Similarly, no child was found infected with three intestinal helminth species when examined with the single SAF-concentration method alone. However, 1 child was found positive for three different helminth species when diagnosis was made based on only the single Kato Katz method.

### Intensity of intestinal helminth infections

The mean egg per gram of *S. manosni*, *A. lumbricoides*, *H. nana*, Hookworm and *T. trichiura* infections observed were 153.6 (Range: 24 to 624), 3032 (Range: 24 to 8400), 318 (Range: 24, 912) and 113.3 (Range: 72, 148), 66.7 (Range: 24, 240), respectively. Out of 31 children positive for *S. mansoni* based on Kato Katz, 48.4% had light intensity infection, 41.9% had moderate intensity of infection and 9.1% had heavy intensity infection. Out of 13 children positive for *A. lumbricoides*, 69.2% had light intensity of infection and 30.8% had moderate intensity of infection. The intensity of hookworm and *T. trichiura* infections in all children positive for the parasites was light. The intensity of *S. mansoni* significantly increased with an increase in age of the children. However, the intensity of *S. mansoni* infection was comparable between males and females. The intensity of *T. trichiura* and *A. lumbricoides* infections were also similar among the different age groups and sex groups.

## Discussion

Knowing the distribution and extent of intestinal parasitic infection in a given community is a prerequisite for planning and evaluating intervention programs. The present study assessed the prevalence of intestinal helminth infections among under-fives children in Wonji Sugar Estate. The prevalence of helminth infection observed among the study participants was 24.3%. *H. nana*, *S. mansoni* and *A. lumbricoides* infections were relatively common among children. Close to 33% of the infected children had at least 2 helminth species. The prevalence of infection increased with an increase in age of the children and it was similar when determined using the Kato Katz and the SAF methods.

The prevalence of helminth infections observed in the current study is lower than a similar report from Tigray [13], Wondo Genet [14] and Methara [15] but higher than a report from Butajira [8]. One of the reasons for the low prevalence of intestinal schistosomiasis (8.8%) compared to the prevalence observed in Tigray Region (1%) [13] might be due to differences in the source of infection.

The prevalence of *S. mansoni* infection observed in the current study is also lower compared to similar previous reports in different regions of the country [14], [15], Uganda [18] and Sierra Leone [19]. This could be due to differences in the geographical areas and water sources, among other things. In the current study the intensity of *S.mansoni* infection documented was also light. This could be due to the periodical cleaning of the irrigation canals and Endod (*Phytolacca dodecandra*) application to kill the snail hosts, *Biomphalaria pfeifferi*. Wonji Sugar Estate.

*S. mansoni* infection showed significant association with age among under five children in the current study where the highest prevalence was observed in the age category 4.1–5.0. This could be due to the playing habit of children in cercariae infested water. Children of age 4.1 to 5 years will more likely play alone in soils and access to water will be more in this age group compared to other age groups.

Despite the relatively high prevalence of *H. nana* and *S. mansoni* infection observed, a lower prevalence of *A. lumbricoides*, hookworm and *T. trichiura* infections were observed among children in the present study. This could be due to the ongoing deworming program of under-fives by the Ministry of Health (MoH) in the study area. On the other hand, the relatively high prevalence of *H. nana* and *S. mansoni* infections observed in under-five children in the area is expected in view of the absence of preventive chemotherapy with praziquantel. Thus, including praziquantel treatment in the deworming program as per the World Helath Organization guidelines would be vital to reduce the burden of these diseases in the study [20].

The Kato Katz and the SAF methods showed significant difference in estimating the prevalence of *S. mansoni* and *H. nana* infection among the current study participants. The Kato-Katz method was superior in estimating the prevalence of *S. manosni* infection and the SAF method was better in determining prevalence of *H. nana* infection. The prevalence of *T. trichiura* infection determined via the Kato Katz method was also significantly higher than the prevalence computed based on results of examination using the SAF method. As a result, the prevalence of intestinal helminth infection with any species among under-fives in the current study was significantly higher when estimated via both methods than when made using either methods alone. The prevalence of multiple infections with any 2, 3 or 4 intestinal helminth species was also estimated better when made based on results of both methods than when either methods were used independently. Thus, using the Kato Katz and the SAF method together would be recommended for better estimation of the prevalence of intestinal helminth infection in under-five children or communities where intensity of infection is light. The Kato Katz method might miss to diagnosis infection with *H. nana* particularly when intensity of infection is low and the SAF method could underestimate low intensity *S. mansoni*, *T. trichiura* and other soil transmitted infections. However, further studies would be required to arive at a conclusion on the performance of both methods on diagnosis of different intestinal helminth species at moderate or heavy intensity of infection.

In conclusion, the present study shows that intestinal helminth infections, particularly *S. mansoni* and *H. nana*, are relatively prevalent among under-five children in the study area. In addition to preventive chemotherapy, provision of safe water supply and latrines, improvement of sanitation and health education on personal and environmental hygiene are indicated to control and eventually eliminate intestinal helminth infections in the area.
